# Changing Pattern of *Chlamydia trachomatis* Strains in Lymphogranuloma Venereum Outbreak, France, 2010–2015

**DOI:** 10.3201/eid2211.160247

**Published:** 2016-11

**Authors:** Olivia Peuchant, Arabella Touati, Clément Sperandio, Nadège Hénin, Cécile Laurier-Nadalié, Cécile Bébéar, Bertille de Barbeyrac

**Affiliations:** University of Bordeaux, Bordeaux, France (O. Peuchant, A. Touati, C. Sperandio, N. Hénin, C. Laurier-Nadalié, C. Bébéar, B. de Barbeyrac);; Institut National de la Recherche Agronomique, Bordeaux (O. Peuchant, A. Touati, C. Sperandio, N. Hénin, C. Laurier-Nadalié, C. Bébéar, B. de Barbeyrac);; Centre Hospitalier Universitaire de Bordeaux, Bordeaux (O. Peuchant, C. Bébéar, B. de Barbeyrac)

**Keywords:** lymphogranuloma venereum, *Chlamydia trachomatis*, *omp*A, L2b genotype, L2 genotype, L2b ompA variants, France, sexually transmitted infection, sexually transmitted disease, proctitis, men who have sex with men, HIV co-infection, outbreak, outbreak strains, bacteria, anorectal lymphogranuloma venereum, MSM, STI, HIV/AIDS and other retroviruses

## Abstract

We describe a change in the molecular epidemiology of *Chlamydia trachomatis* strains involved in an outbreak of rectal lymphogranuloma venereum in France during January 2010–April 2015. Until 2012, the *C. trachomatis* L2b strain predominated; however, starting in 2013, most cases involved the L2 strain. We also identified 4 genetic L2b *omp*A variants.

Lymphogranuloma venereum (LGV) is a sexually transmitted infection caused by the invasive L genovars of *Chlamydia trachomatis*. Since 2003, outbreaks of LGV have spread across Europe and other high-income countries, mainly among HIV-infected men who have sex with men (MSM) (*1*–*5*). Almost all LGV cases have been caused by the *C. trachomatis* L2b variant, which harbors an A/G substitution on the *omp*A gene at position 485, suggesting a single source of origin for the outbreaks (*6*,*7*). Recently, however, 2 *C. trachomatis* variants co-circulated during an LGV epidemic in Spain (*8*). The objective of this study was to investigate if genetic variations exist within LGV *C. trachomatis* strains circulating in France during 2010–2015 by sequencing of the *omp*A gene.

## The Study

In January 2010, France introduced sentinel surveillance for *C. trachomatis* proctitis, approved by the country’s Data Protection Authority, to monitor the LGV outbreak. Laboratories perform routine testing for *C. trachomatis*, and positive anorectal specimens are referred to the French National Reference Center for Chlamydiae (Bordeaux, France) for LGV testing, using a real-time PCR targeting a 36-bp deletion in the *pmp*H gene (*9*). For each patient, clinical, biological, and sexual behavior data are collected after written consent is obtained.

During January 2010–April 2015, we retrospectively selected 179 LGV-positive anorectal specimens from the surveillance samples. To ensure that samples were representative of infections in the LGV-infected population, we used the following criteria for selecting samples for each year of the study: 1) the percentage of specimens analyzed each year was the same and corresponded to ≈12% of anorectal LGV cases diagnosed in France per year; 2) the residences of patients were classified as in the Paris area versus other regions of France to respect the geographic distribution of cases during each year of the survey; 3) samples were distributed over the 12 months of each year, except 2015; and 4) specimens had a high bacterial load, defined as a cycle threshold of <30 using the *pmp*H real-time PCR. We analyzed 23 *C. trachomatis* specimens from 2010, 24 from 2011, 24 from 2012, 40 from 2013, 49 from 2014, and 19 from January–April 2015.

Amplification of the *omp*A gene was performed directly on specimens. A 1,100-bp fragment was amplified by nested PCR, using the NLO and NRO primers and PCTM3 and SERO2A primers (*10*), and sequenced in both directions. An L genovar was confirmed for all specimens by *omp*A sequencing. Most specimens (52.5%, 94/179) had *omp*A sequences identical to that of the *C. trachomatis* L2b/UCH-1/proctitis reference strain (GenBank accession no. AM884177.1); 61 (34%) specimens had sequences identical to that of reference strain L2/434/BU (GenBank accession no. AM884176.1). In the remaining 24 specimens, we identified 4 genetic L2b *omp*A variants that had nonsynonymous single-nucleotide polymorphisms, compared with nucleotide sequence of the L2b/UCH-1/proctitis reference strain. One variant, L2b *omp*A variant 1 (designated L2bV1; GenBank accession no. JX971936), was found in 19 specimens and featured a C→A substitution at position 517 (Leu173Ile) (*8*,*11*). A second variant, L2bV2 (GenBank accession no. KU518893), was detected in 1 specimen and had an A→C substitution at position 515 (Lys172Thr). The third variant, L2bV3 (GenBank accession no. KU518894), was detected in 2 specimens and shared a C→A substitution at position 493 (His165Asn). The fourth variant, L2bV4 (GenBank accession no. KU18892), was detected in 2 specimens and featured a C→A point mutation at position 286 (Ala96Thr).

Our analysis of the distribution of the *C. trachomatis* *omp*A genotype during 2010–2015 showed that the percentage of L2b strain was significantly lower in 2013 (35%, 14/40), 2014 (38.8%, 19/49), and 2015 (31.6%, 6/19) than in 2010 (86.9%, 20/23), 2011 (70.8%, 17/24), and 2012 (75%, 18/24) (p<0.05 by χ^2^ test) (Figure). Conversely, the percentage of L2 strain was significantly higher in 2013 (52.5%, 21/40), 2014 (44.9%, 22/49), and 2015 (52.6%, 10/19) than in 2010 (4.3%, 1/23), 2011 (20.8%, 5/24), and 2012 (8.3%, 2/24) (p<0.05 by Fischer exact test). The percentage of L2b *omp*A variants was similar during each year of the survey (Figure). In 2012 and 2013, the distribution of L2 and L2b strains and L2b *omp*A variants was the same in Paris and other regions of France, showing that the shift was not due to regional variation (p = 0.86 by χ^2^ test).

**Figure:**
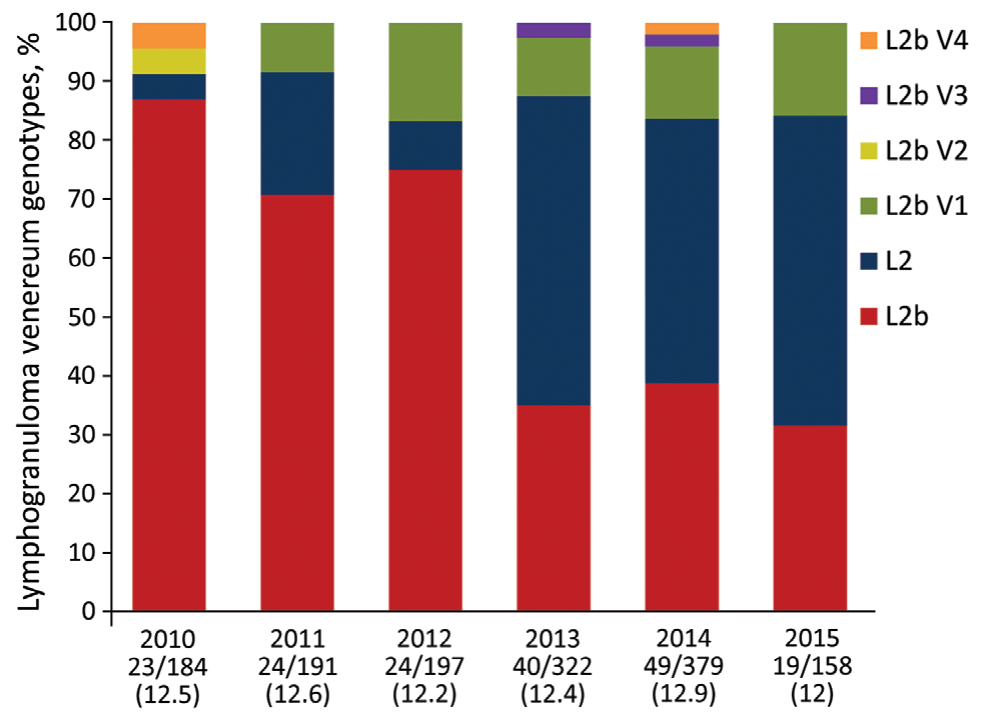
Distribution of lymphogranuloma venereum–associated *Chlamydia trachomatis* genotypes and variants, according to sequencing results of the *omp*A gene of representative patient samples, France, 2010–2015. Numbers below bars indicate no. samples analyzed/no. cases total (%).

All specimens were from men. Symptoms (rectal syndrome, rectal pains, anal discharge, rectal bleeding) were present in all cases, and clinical features were similar. Data about sexually transmitted bacterial co-infections (e.g., *Neisseria gonorrhoeae*, syphilis) were available for 64.2% (115/179) of patients and showed a similar prevalence among those infected with L2b (34.6%, 18/52), L2 (35.3%, 12/34), or L2b *omp*A variants (38.5%, 5/13). Serologic HIV status was documented for 112 patients; results were positive for 86.8% (33/38), 79.7% (47/58), and 68.8% (11/16) of the patients carrying L2 strain, L2b strain, or L2b *omp*A variants, respectively (p = 0.14). The percentage of MSM was lower among patients infected with L2b *omp*A variants (76.9%, 10/13) than among those infected with L2 (100%, 24/24) or L2b strains (95.7%, 45/47) (p = 0.063). Having an occasional, versus a steady, sex partner was frequently reported, ranging from 76.5% (26/34) in the L2 group to 100% (8/8) in the L2b *omp*A variants group.

## Conclusions

We describe a change in the molecular epidemiology of *C. trachomatis* strains involved in an outbreak of anorectal LGV in France. Our results show that, until 2012, the L2b strain predominated, a finding that is in agreement with reports coming from other countries in Europe (*12*). However, L2b is not a recently emerged strain: the strain was present in 1981 among MSM in San Francisco, California, USA (*12*). Different LGV strains have spread widely in the MSM community since 2013, and prevalence of the L2 genotype has increased. These data suggest that the co-circulation of the 2 predominant LGV strains could be the result of 2 independent introductions. The L2 strain is more prevalent in the United States (*13*), and the last known outbreak was in 1992 in the Caribbean area (*14*). Knowing the country of residence of the partner who was the possible source of infection would potentially enable identification of distinct introduction pathways for L2 and L2b strains.

Our findings show that 4 genetic *C. trachomatis* L2b *omp*A variants have been circulating in France since 2010. Three of the variants have amino acid changes in the variable domain II, which has been described as a common antigenic domain for *C. trachomatis*. The L2bV1 strain was identified during September 2011–March 2012 in 4 specimens collected in New York, USA, and in 1 specimen collected in Spain between 2009 and 2011 (*8*,*11*). In our study, *C. trachomatis* strain L2bV1 was first identified in 2011 and then each year thereafter; it was the predominant L2b *omp*A variant. The 3 other variants had not been previously described.

Patient characteristics did not differ with regard to clinical data, sexual behavior, or the *C. trachomatis* genotypes involved in LGV infections. Our results are discordant with those of Rodríguez-Domínguez et al. (*8*), who showed less aggressive symptoms among patients infected by *C. trachomatis* L2 strain than those infected by L2b strain.

Our results must be confirmed by genetic characterization of more specimens. However, we observed genetic diversity of LGV *C. trachomatis* strains when testing as few as 12% of reported anorectal LGV patients a year. Future research might examine if the increase of LGV cases in other countries is also associated with an increase in *C. trachomatis* L2 strain.
